# Targeting immunoglobulin E in atopic dermatitis: A review of the existing evidence

**DOI:** 10.1016/j.waojou.2021.100519

**Published:** 2021-03-19

**Authors:** Andreas Wollenberg, Simon Francis Thomsen, Jean-Philippe Lacour, Xavier Jaumont, Slawomir Lazarewicz

**Affiliations:** a Department of Dermatology and Allergy, Ludwig-Maximilian-University, Munich, Germany; b Department of Dermato-Venereology and Wound Healing Centre, Bispebjerg Hospital, University of Copenhagen, Copenhagen, Denmark; c Department of Dermatology, Archet Hospital, Université Côte D'Azur, Centre Hospitalier Universitaire Nice, Nice, France; d Novartis Pharma AG, Basel, Switzerland

**Keywords:** Apheresis, Atopic dermatitis, Biologics, Immunoglobulin E, Omalizumab

## Abstract

Immunoglobulin E (IgE) plays an essential role in many allergic diseases. This review highlights the role of IgE in atopic dermatitis (AD), a common, chronic, and complex skin inflammation, and the available therapeutic approaches that target IgE in AD. We examine the existing data showing the use of omalizumab, the only biologic anti-IgE therapy available in clinical use, plasma apheresis, and a combination of both therapeutic approaches for the treatment of AD. Existing data on the efficacy of omalizumab in AD are inconclusive. A limited number of randomised controlled studies, few uncontrolled prospective and retrospective reports, as well as multiple case series and case reports observed varying degrees of the efficacy of omalizumab in AD. Omalizumab displays a trend of higher efficacy in AD patients with low IgE levels compared with those with very high-to-extremely high serum IgE concentrations. Plasma apheresis and its combination with omalizumab show good efficacy, even in patients with unusually high serum IgE concentrations. Combining apheresis and anti-IgE treatment may serve as a comprehensive therapeutic approach for patients with elevated levels of IgE. Dedicated clinical studies with robust study designs are needed to establish the therapeutic efficacy of omalizumab in AD.

## Introduction

Atopic dermatitis (AD) is a common, chronic, and complex skin inflammation that involves a combination of genetic factors affecting the skin barrier, environmental factors, and immunological response. AD affects nearly 15–20% of children and 1–3% of adults, with 95% of those affected experiencing an early onset below the age of 5.^1^^,^^2^ It is estimated that within the past few decades, the incidence of AD may have increased 2–3 fold in the industrialised world.^1^ The prevalence of AD varies with an estimated 230 million patients affected worldwide.^3^ Recent data show a 2.2–8.1% prevalence across Europe and the United States.^4^^,^^5^ Similarly, data from Asia show an increasing prevalence in countries such as India and China.^3^

Typically, AD is most common in young children and resolves before adulthood. However, the presence of severe AD with multiple factors such as early onset of disease, filaggrin gene (*FLG*) mutations, food allergies, and sensitization may result in persistence of the condition into adulthood.^6^ About 50% of the patients may develop other allergic symptoms within the first year of life, and ~60% of children affected with AD develop other atopic co-morbidities in a process that has been defined as the atopic march.^1^ Atopic manifestations with characteristic sequential immunoglobulin E (IgE) antibody responses appears early in life, persists over many years, and may remit spontaneously with age.^6^ However, the existence of the atopic march has been controversial with suggestions that evidence is based on population level rather than on symptom profile at the individual level.^7^ Nevertheless, evidence suggests that expression of AD early in life is a risk factor for developing allergic rhinitis and asthma later in life, and therapies targeting the prevention of AD by repair of the epidermal barrier may prevent subsequent events of rhinitis or asthma.^6^^,^^8^ AD is a predominantly T helper 2 cell (Th2)-driven disease with more localised symptoms during childhood, that may develop into a systemic disease, manifesting as numerous co-morbidities in adulthood.^9^^,^^10^ The prevalence of asthma in patients with AD ranges between 14.2 and 52.5%, and one-third of all children with eczema may develop asthma in childhood.^11^ Allergen-specific IgE sensitization is a key feature of extrinsic AD; a mandatory role in the pathogenesis remains to be established.^12^ The role of IgE in AD and appraisal of the existing evidence on targeting IgE for treatment is discussed in this review.

## Role of allergy and IgE in the pathophysiology of AD

IgE plays a central role in allergen-induced inflammatory processes in various atopic diseases and presents a viable target for therapy. IgE binds to various immune cells by high-affinity IgE receptors (FcεRI), which differ in the presence or absence of a beta-chain, and acts as both an effector for chemical mediator release and regulator for cytokine production.^13^ Signalling in mast cells and basophils is followed by the release of preformed inflammatory mediators, whereas Langerhans cells and inflammatory dendritic epidermal cells use this receptor for IgE-mediated internalisation of antigens for antigen presentation.^14^ The IgE-FcεRI mediated antigen presentation primes T cells within the lymphatic system, leading to the expansion of activated Th2 cells and allergic inflammation. IgE bound to FcεRI on dendritic cells also acts as immune surveillance during steady state.^15^ The roles of other IgE-receptors, FcεRII/CD23, and the epsilon binding protein identified on skin dendritic cells are less clear. In summary, there is considerable evidence demonstrating a pivotal role of allergen-specific IgE in AD and a possible mechanism through an auto-allergic IgE cascade.

AD can be dichotomised into intrinsic and extrinsic forms. Intrinsic AD presents with normal serum levels of total and specific IgE, a female predominance, and a relatively preserved skin barrier function.^16^ The extrinsic phenotype constitutes ~80% of all children with AD, and is driven by skin barrier function abnormalities, sensitization, and high-to-extremely high levels of IgE (≥20,000 IU/mL). *FLG* mutations, leading to conditions such as ichthyosis vulgaris and palmar hyper-linearity, constitute ~27% of patients with extrinsic AD. These patients have a higher probability of comorbid asthma and allergic rhinoconjunctivitis, show higher counts of blood eosinophil, have higher severity of the disease, and a lower quality of life (QoL) compared with patients with intrinsic AD.^17^ Pugliarello et al proposed different clinical variants of AD according to the time of onset of the disease, morphology and localisation of dermatological symptoms, the involvement of IgE in pathogenesis, AD associated with specific symptoms, and persistence of symptoms.^18^ More recently, based on the onset and course of the disease, 4 phenotypes of AD associated with or without food allergy were proposed: early transient, early persistent, late onset, and infrequent. Patients with very early onset of the disease and persistent symptoms were more likely to present with more severe disease and a strong association with asthma and food allergy.^19^ Food allergen sensitization and food allergy were demonstrated in young and older children to be associated with *FLG* loss-of-function linked skin barrier impairment.^20^

**Fig. 1:**
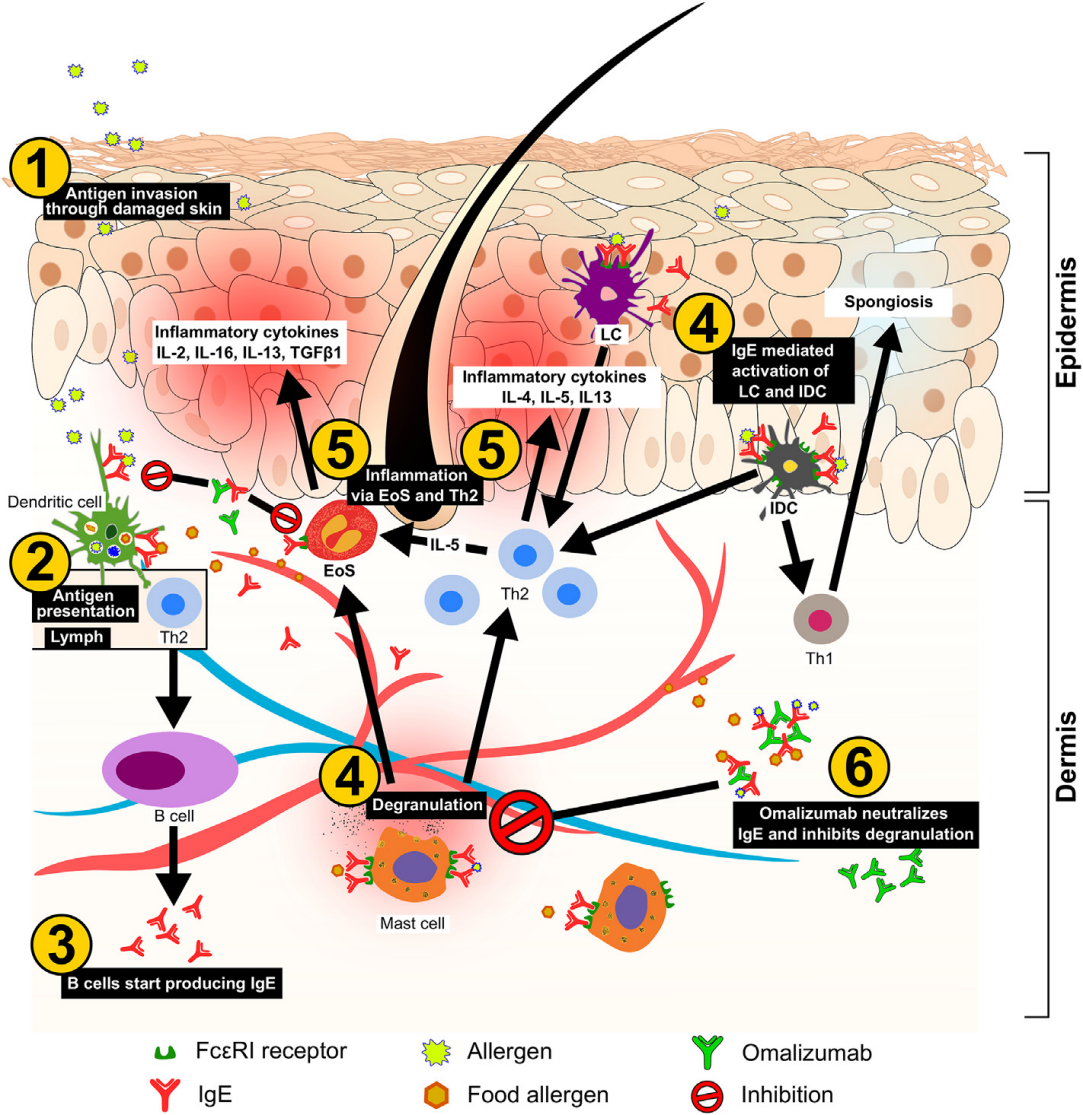
Simplified mechanism of AD and the role of IgE. Antigen penetration through damaged skin and presentation via APCs leads to a Th2 response. The resulting IgE production against the antigen can lead to degranulation of mast cells in the presence of the external antigen or food allergens causing a local inflammatory response and recruitment of other inflammatory cells such as EoS. LCs and IDCs activated via recognizing pathogen-derived antigens, promote Th1 and Th2 driven immune responses in acute AD lesions. Omalizumab, by neutralizing IgE, can inhibit mast cell degranulation and dendritic cell activation. AD, atopic dermatitis; EoS, eosinophils; IDC, inflammatory dendritic cells; IgE, immunoglobulin E; IL, interleukin; LC, Langerhans cells; Th2, T helper cell.

Skin barrier disruption increases its permeability to external antigens/allergens and facilitates Th2 immune response through antigen-presenting cells such as Langerhans and dendritic cells. This leads to an increase in the production of IgE, which mediates subsequent hypersensitivity responses to antigen exposures (Fig. 1). Auto-IgE antibodies to resident self-antigen, or anti-IgE and anti-FcɛRI in intrinsic atopy may follow a similar cascade.^21^ Hence, IgE plays an important role in the pathogenesis of AD, and is present at increased levels both in the serum and skin of patients. There is a significant association between higher levels of IgE and the severity of AD.^17^ However, even patients with normal levels of total serum IgE may show a positive skin prick test and many AD patients have low levels of IgE.^22^ This emphasizes the importance of specific IgE rather than the total IgE, even though a positive correlation exists between them.^23^

## Current treatment recommendations for AD

Current treatment options for AD include avoidance of identified allergenic or non-allergenic triggers, barrier-improving emollients, non-specific anti-inflammatory and immunosuppressive therapies, such as topical class II glucocorticosteroids and calcineurin inhibitors, for patients with mild AD or transient eczema.^24^, ^25^, ^26^, ^27^ Patients with moderate AD or recurrent eczema, based on the **scor**ing **a**topic **d**ermatitis (SCORAD) index of 25–50, are recommended topical tacrolimus or class II/III topical glucocorticosteroids in combination with the liberal use of emollients and frequent follow-up clinical examinations to help reduce relapses. Non-pharmacological therapies for patients may include ultraviolet phototherapy, wet wrap and climate therapy, and psychosomatic counselling.^24^ Severe AD with persistent eczema, with a SCORAD index of >50, needs systemic immunosuppressive treatments, such as cyclosporine A, a short course of oral glucocorticosteroids, methotrexate, azathioprine, mycophenolate mofetil, and photochemotherapy with psoralen and ultraviolet A or treatment with a Th2-blocking biological drug such as dupilumab.^25^ Due to inadequate evidence and for important safety reasons, patients and especially children with severe AD have even more limited therapeutic options. Dupilumab, a monoclonal anti-IL-4Ra blocking the action of IL-4 and Il-13, is currently the only licensed biologic for the treatment of adult patients with moderate-to-severe AD and for adolescent patients aged ≥12 years who are candidates for systemic therapy.^24^^,^^25^ Presently, other biologics are not indicated for the treatment of AD due to insufficient proof of efficacy. Nevertheless, their use may serve as an exploratory alternative to achieve response in patients with severe AD refractory or intolerant to other recommended treatments, and offer insights into the pathogenic mechanisms in AD.^25^ Several biologics, such as nemolizumab (anti-IL-31), lebrikizumab (anti-IL-13), tralokinumab (anti-IL-13), and ustekinumab (anti-IL-12/23), are currently in development for AD.

Omalizumab is a humanised monoclonal anti-IgE antibody which is approved for the treatment of severe allergic asthma, chronic spontaneous urticaria (CSU), and chronic rhinosinusitis with nasal polyps.^28^ Blocking IgE and consequently mast cell and basophil activation in the allergic cascade has been the therapeutic strategy behind the use of omalizumab in atopic diseases such as allergic asthma and AD. Omalizumab is the only anti-IgE antibody on the market with over 15 years of clinical experience and over 1.3 million patient-years of exposure (PSUR: Novartis Data on File as of December 31, 2019). Omalizumab forms complexes with IgE by binding to its Cε3 domain. The binding action of omalizumab with free IgE reduces serum levels, resulting in an anti-inflammatory effect. Omalizumab-IgE complexes may also act to capture any free antigen that can trigger an immune response (Fig. 1). Omalizumab has been investigated as a therapeutic option in various IgE-mediated allergic diseases such as allergic asthma, food allergy and allergic rhinitis, as well as non-allergic diseases such as chronic rhinosinusitis with nasal polyps and CSU. Many studies have investigated the role of IgE and the efficacy of omalizumab for the treatment of various dermatological conditions. AD is an actively explored disease area for IgE-targeted treatment. However, the current indication of omalizumab does not include treating AD patients.^28^ Besides anti-IgE therapy using omalizumab, plasma apheresis has also been explored as an innovative therapeutic option targeting IgE in patients. Treatment algorithm combining apheresis and anti-IgE treatment with omalizumab has also been explored.^29^

## Anti-IgE therapy in atopic dermatitis

**Fig. 2:**
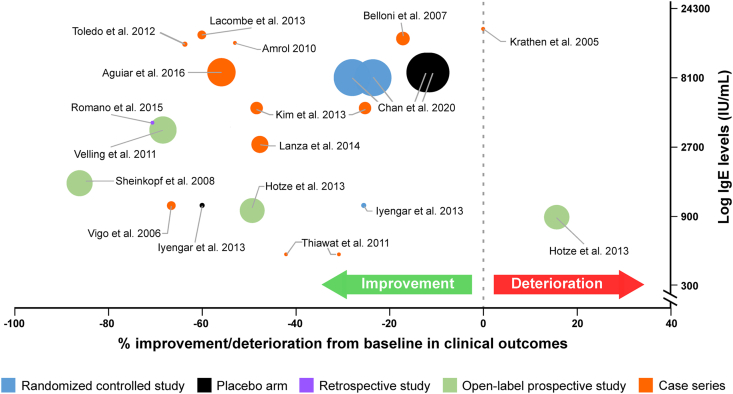
Patient population, their baseline IgE levels, and corresponding clinical outcome of treatment of AD with omalizumab in terms of percentage change from baseline in SCORAD/DLQI/Pruritus/EASI scores in various studies. Size of the dots are proportional to the number of patients analysed. Hotze et al., 2013 showing deterioration consists of all patients with *FLG* mutations DLQI, dermatological life quality index; EASI, Eczema Area and Severity Index; *FLG*, filaggrin gene; IgE, immunoglobulin E; SCORAD, scoring atopic dermatitis.

Currently, omalizumab is the only approved anti-IgE therapy available for clinical use. Most evidence for the use of omalizumab in AD has been generated through numerous case studies and a few case series. For the preparation of this article, an Ovid literature search was conducted with Medline, Embase, and Biosys as the databases, on anti-IgE treatment in AD from January 2002 to May 2020 The search terms used included omalizumab, anti-IgE, atopic dermatitis, allergic dermatitis, and atopic eczema. Up until May 2020, 3 placebo-controlled studies, 4 prospective studies, 3 retrospective studies, and 33 case studies/series have investigated the use of omalizumab in AD (Fig. 2 and Table 1).

**Table 1:**
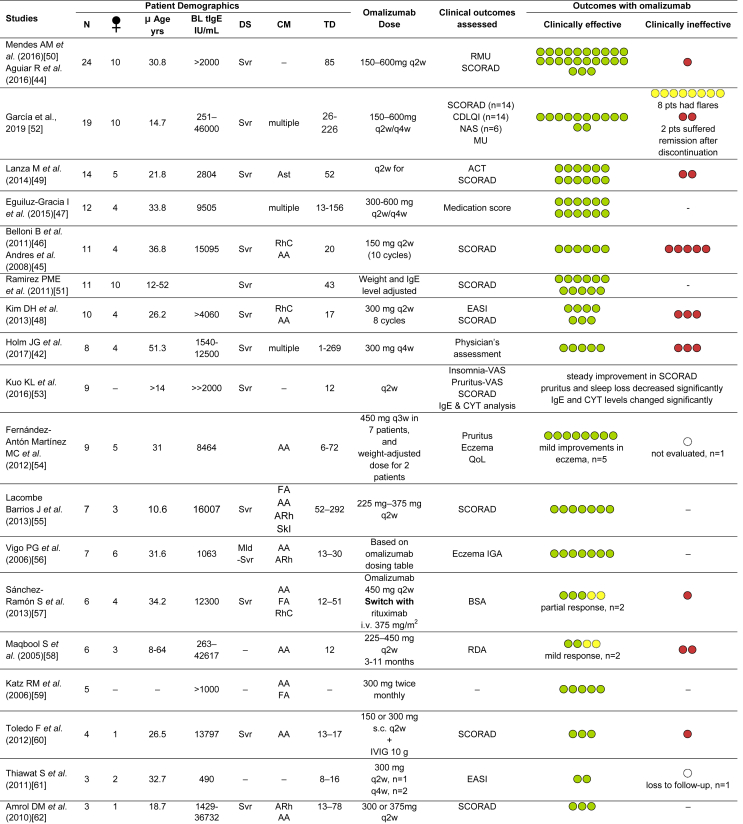

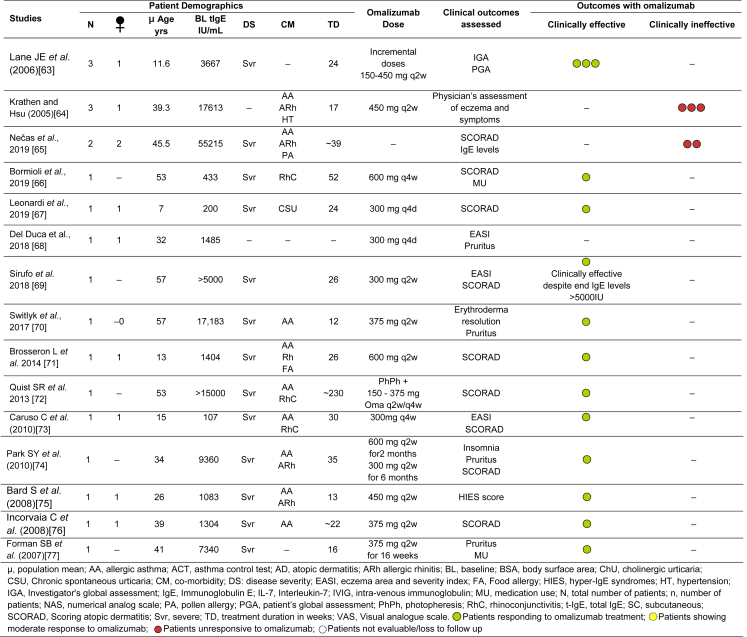
Evidence of efficacy of omalizumab in AD from Case Series and Case Reports.

### Randomised controlled studies

Three randomised, controlled pilot studies have explored the efficacy of omalizumab in a small number of paediatric and adult patients.^30^, ^31^, ^32^ Besides investigating the clinical outcome of omalizumab treatment in patients, a study tested the hypothesis that anti-IgE therapy may modulate Th2 response in patients. The study investigated the efficacy of omalizumab in 8 paediatric and adult patients (between 4 and 22 years of age) with high baseline IgE levels, over 24 weeks of treatment, and showed 20–50% improvement in the SCORAD index and 50–75% reduction in thymic stromal lymphopoietin in all patients treated with omalizumab. A decrease in levels of thymus and activation regulated chemokine (TARC), tumour necrosis factor (TNF) receptor superfamily member 4 (OX40L), and IL-9. An increase in the regulatory cytokine IL-10 was also observed in patients treated with omalizumab.^32^ However, the placebo response was strong and showed a SCORAD improvement ranging from 45 to 80%. This lack of resolution of clinical response between the two arms, despite the significant difference in the effect on the cytokine environment, could be a result of the extremely small sample size of 4 patients in each arm contributing to the difference in patient demographics and disease characteristics.^32^ The other randomised controlled study investigated the expression of IgE and its receptors on cells and serum components in 20 patients after 16 weeks of treatment with omalizumab.^31^ Interestingly, although no change was observed in the level of free serum IgE or IgE-receptor saturation, a noticeable decrease in the surface-bound IgE and surface expression of FcεRI receptors on basophils, monocytes, and dendritic cells was evident in the placebo group. This is in agreement with the stark placebo effect observed in some patients with AD. Pruritic assessment showed that the severity of symptoms increased in the omalizumab group compared with placebo. The investigators’ global assessment (IGA) of the disease was similar between the groups. The authors reason that this may be reflective of either a limited distribution of omalizumab into the outer skin leading to residual levels of IgE sufficient to elicit a response, or a higher constitutive expression of FcεRI receptors in the skin compared with blood. The authors speculate that the effect of omalizumab in AD may be more evident in acute forms of the disease, which is dominated by Th2/Th17 cytokine milieus sustained by IgE.^31^ The effect of placebo on itch and other symptoms of AD is well documented.^33^^,^^34^ Shorter studies with a proper blinding design and reduction of confounders, such as concomitant medications, may help reduce false positives.^34^ Nevertheless, omalizumab treatment reduced the density of surface-bound IgE and IgE-receptors and showed favourable effects on inflammatory biomarkers such as TSLP, OX40L, TARC, and IL-10.^31^^,^^32^ The Atopic Dermatitis Anti-IgE Paediatric Trial (ADAPT) was a randomised, double-blind study, that evaluated the possible role of omalizumab (>600 mg every 2 or 4 weeks) in the management of severe paediatric AD for 24 weeks.^30^ The primary objective of the study was the change in objective SCORAD after 24 weeks of treatment. The study showed that omalizumab significantly reduce disease severity and improve QoL in paediatric patients with severe AD and highly elevated IgE levels (median baseline total IgE of 8373 IU/L) compared with placebo. Objective SCORAD index dropped from 55.5 at baseline to 43.1 at Week 24, achieving the minimum clinically important difference 8.5 in the omalizumab group. In comparison objective SCORAD index decreased only by 5.1 for the placebo group. Significant improvements were observed in the eczema area and severity index score at Week 24 in the omalizumab group; the median days of topical corticosteroids was 48% higher in the placebo group versus patients treated with omalizumab.^30^

### Observational studies

Several open-label prospective and retrospective studies have investigated the efficacy of omalizumab, at doses 150–450 mg every 2 weeks or every 4 weeks, for 28–168 weeks.^29^^,^^35^, ^36^, ^37^, ^38^, ^39^ All studies investigated patients with very high mean baseline IgE levels ranging between 883 and 4243 IU/mL. Clinical outcomes measured including SCORAD, IGA index, and dermatological life quality index (DLQI) showed a baseline IgE-dependent response. Patients with lower baseline IgE show a positive response to treatment with omalizumab compared with patients with very high-to-extremely high serum IgE (Fig. 2). This is contrary to what is observed in patients with CSU.^40^ However, good–partial responses were observed in patients with very high IgE levels, suggesting that the mechanism of omalizumab therapy in AD may be more complex than just neutralizing IgE.^37^, ^38^, ^39^ Response rates in different AD studies ranged between 40 and 100% of patients showing positive clinical outcomes.^29^^,^^35^, ^36^, ^37^, ^38^, ^39^ In a small group of patients, omalizumab treatment was associated with lower use of oral corticosteroids, even if using the latter is discouraged according to guidelines, especially in paediatric AD.^25^^,^^39^ Interestingly, one study showed that patients with a *FLG* mutation were unresponsive to omalizumab treatment, suggesting that the presence of primary skin barrier deficiency may likely be a factor for non-response.^35^^,^^41^ In disagreement, Holm et al reported 1 patient in their study with *FLG* mutation showing a positive response, while 2 patients without the mutation showed no response to omalizumab treatment.^42^ A retrospective analysis of 10 patients who were treated with 600 mg omalizumab showed complete response (patients able to discontinue omalizumab and stay controlled with standard dose of antihistamines) in 5 patients, and partial response (patients able to stay controlled with add-on omalizumab) in the remaining. Of the 5 complete responders, 4 were recorded to have undergone remission after treatment withdrawal.^43^ These results were based on the outcomes on patients’ medical records and not on clinical evaluation tools, hence, should be interpreted with caution.

### Case series and case reports

Definite conclusions may not be drawn from reported cases, as there could be confounding factors present, such as concomitant medications, comorbid conditions, or other unreported events that may influence outcomes. In addition, many case reports incline to describe extreme and rare forms of the disease, which may not represent the general patient population. Nevertheless, case series and case reports can serve as proxies to the rigorous investigative approach applied in large, well-designed controlled studies missing here. They help in generating hypotheses and identifying appropriate patient characteristics for larger studies. They can also identify responses and outcomes in special patient populations such as pregnant women, the elderly, children, and patients with comorbidities such as cancer, human immunodeficiency virus, or other chronic diseases.

The use of omalizumab in patients with AD has been presented in multiple case series and case reports.^42^^,^^44^, ^45^, ^46^, ^47^, ^48^, ^49^, ^50^, ^51^, ^52^, ^53^, ^54^, ^55^, ^56^, ^57^, ^58^, ^59^, ^60^, ^61^, ^62^, ^63^, ^64^, ^65^, ^66^, ^67^, ^68^, ^69^, ^70^, ^71^, ^72^, ^73^, ^74^, ^75^, ^76^, ^77^ Details on these cases are summarised in Table 1. Patients across regions with average age ranging between 7 and 51 years, extremely variable levels of baseline IgE, and omalizumab (150–600 mg q2w or q4w) treatment duration between 8 and 292 weeks, have been studied. The majority of studies report a high proportion of patients, with varying demographics and disease characteristics but mostly high-to-very high IgE levels, exhibiting improvements in the SCORAD index after omalizumab treatment.^44^, ^45^, ^46^, ^47^, ^48^, ^49^, ^50^, ^51^ In various case series studying ≥10 patients, a cumulative total of 83 patients out of the 101 patients showed clinical efficacy with omalizuamb. The largest case series reporting 24 patients with AD treated with omalizumab, all except one were reported to have improved.^44^^,^^50^ Garcia et al reported a series of evaluation in 19 patients and reported 85.7% patients moved from severe to mild or moderate disease severity at the end of treatment.^52^ Among 14 AD patients comorbid with asthma, 12 showed significant reduction in severity of both asthma and AD after q2w omalizumab therapy for 1 year.^49^ Belloni et al reported data from 11 AD patients treated with omalizumab for 20 weeks, showed good clinical response in 6 patients. Two patients showing insignificant change from baseline in SCORAD, while the remaining 3 showed worsening of symptoms.^46^ In a study involving 10 AD patients with allergic co-morbidities, 7 patients responsive to omalizumab treatment did not experience worsening of their eczema symptoms while on omalizumab and up to 4 months after the treatment ended.^48^ Nevertheless, there were 3 patients who showed non-responsiveness and had worsening of the disease while on therapy.

## Apheresis targeting IgE

Therapeutic plasma exchange (TPE) or plasmapheresis is a non-specific extracorporeal blood purification technique used to remove circulating pathogenic immune factors such as cytokines, autoantibodies, immunoglobulins and immunocomplexes. In recent years, different plasmapheresis techniques have emerged as a novel and innovative method to treat various immunological diseases, which include plasma exchange, plasma filtration and immunoadsorption. A pilot study in 12 patients that utilised extracorporeal immunoadsorption to deplete all Ig types demonstrated a significant decrease in skin-bound IgE, and significantly reduced disease severity measured with SCORAD as early as 3 weeks from the initiation of the procedure.^78^ Specific adsorption columns with polyclonal sheep antihuman immunoglobulin antibodies binding to all immunoglobulin variants, including IgE, could be used for depleting IgE levels in the blood. However, a non-specific immunoadsorption may encounter severe adverse events related to infections.^78^ Although the mechanism of action in play for AD is elusive, a possible alteration in tissue-bound IgE or alterations in the levels of auto-reactive IgE have been suggested and recently demonstrated.^79^, ^80^, ^81^ Nevertheless, the removal of circulating inflammatory mediators, such as eosinophil, eosinophil cationic protein, soluble interleukin-2 receptor and soluble E-selectin, with non-IgE specific extracorporeal apheresis cannot be ruled out as a possible mechanism of clinical efficacy.^82^ For AD patients with very high-to-extremely high serum IgE, apheresis is possibly the most logical treatment approach. Both non-specific and IgE-specific immunoadsorption has shown encouraging effects in patients unresponsive to standard systemic treatments.^79^, ^80^, ^81^, ^82^, ^83^, ^84^, ^85^, ^86^ These outcomes did not necessarily correlate with decreased levels of serum IgE. On the contrary, serum IgE levels in some patients were observed to be higher after the procedure was discontinued, and in most patients, IgE levels quickly return to near baseline values.^79^^,^^81^^,^^85^ Nevertheless, sustained reduction in tissue levels of IgE has been demonstrated in biopsies after immunoadsorption procedures.^78^^,^^80^ An open-label pilot trial with 11 patients of severe recalcitrant AD showed that peripheral IgE levels can selectively and significantly be reduced using a single-use IgE-selective absorbent column. Desirable clinical response was observed more prominently in patients with baseline IgE ≤6000 IU/mL versus patients with higher baseline IgE levels.^80^ Therapeutic plasma exchange in patients with moderate-to-severe AD, with baseline serum IgE levels in the normal range, demonstrated a small decrease in circulating IgE, and more importantly, considerable improvement in the SCORAD index.^82^^,^^84^ Double-filtration plasmapheresis (DFPP) has also been investigated as a possible therapy for recalcitrant AD unresponsive to standard treatments. Interestingly, even though DFPP did not show any remarkable effect on levels of serum IgE, the procedure significantly improved the SCORAD index as early as 1 week compared with patients who were on standard immunosuppressive therapy. A significant decrease in serum eosinophil cationic protein concentrations was also observed, indicating a suppression of the Th2 response. Even though the reduction in SCORAD was significant, these were clinically low improvements of ~20% and corresponded closely with ~33% reductions in serum IgE immediately after DFPP.^85^ Hence, it could be assumed that DFPP may not be a suitably effective treatment for patients with AD. IgE-specific immunoadsorption has demonstrated to markedly reduce IgE levels in patients with high-to-extremely high levels of baseline serum IgE, accompanied with up to 75% of patients improving ≥50% on the EASI score. Improvements in QoL and symptoms of AD were observed up to 4 months after therapy discontinuation, despite the rapid regeneration of IgE observed after each and final cycle of treatment.^81^ Reich and group, in 2 separate studies, showed that IgE-selective immunoadsorption can reduce peripheral IgE levels by up to 90%, and improve severity of disease and QoL of patients.^79^^,^^80^ Similarly, Kasperkiewicz and group also showed efficacy of IgE-selective immunoadsorption in patients with severe AD and in patients with recalcitrant AD.^87^^,^^88^ The probability of response to IgE-immunoadsorption increased with factors such as high baseline IgE levels and presence of a moderate-to-severe AD being treated with systemic therapy.^79^^,^^80^ Apheresis-induced reduction of serum IgE was accompanied with a reduction in expression of cutaneous IL-13, which correlated well with the clinical response.^80^ Similarly, another study investigating the efficacy of IgE-immunoadsorption in 5 patients demonstrated a notably stronger clinical effect in patients with extremely high IgE levels (>5000kU/L) compared with patients with moderately elevated serum IgE, and reported significant improvements in the SCORAD index of all patients during the study. However, an increase in the SCORAD index was observed after the treatment regimen ended.^86^

## Combination of apheresis and anti-IgE therapy

Most studies investigating the efficacy of omalizumab in AD have been conducted under uncontrolled conditions without omalizumab dose-adjusted for patient's baseline IgE using the standard dosing table. This would be impractical for patients with very high-to-extremely high IgE levels and may partially explain the lack of efficacy observed in some studies. The extremely high IgE levels of patients in these studies may not be completely neutralised even with the highest dose of omalizumab, which increasing further would cause potential concern of adverse events and consequent increase in the cost of treatment. Zink et al investigated a combination of apheresis and omalizumab, showing that in patients with high levels of IgE, an immunoadsorption therapy prior to anti-IgE treatment may serve some clinical benefits.^29^ A notable outcome of the combination therapy was the sustained decrease in total IgE levels observed compared with only transient suppression of IgE levels with immunoadsorption alone.^81^^,^^83^^,^^87^ This treatment regimen may also be supported by the findings from a recent meta-analysis which concluded that omalizumab is more likely to be effective in patients with low serum IgE compared with AD patients with very high levels of serum IgE.^89^ A case study of a 53-year-old man with relapsing severe AD also explored the combination of apheresis and anti-IgE over a period of 3 years. The combination treatment led to a gradual decrease in the levels of IgE and showed a well-controlled disease.^72^ The aforementioned approach to combine apheresis and anti-IgE treatment may become a useful therapeutic regimen for patients with high-to-extremely high plasma levels of IgE,^29^^,^^72^ but needs validation in a larger patient population.

## Discussion

IgE is strongly linked to the pathophysiology of allergic conditions. The Th2 favoured immune environment seen in many allergic conditions is also evident in AD, especially during childhood. IgE directed against external allergens and plausible auto-antigens may be a possible target that exacerbates the disease.^90^ The mechanism of dupilumab, the only biologic approved for treatment of AD, has also been proposed to indirectly affect the production of IgE by blocking the IgE switching cytokines (IL-4 and IL-13) on B-cells. Hence, targeting IgE for management and treatment of patients is expectedly well-reasoned. Omalizumab, the only anti-IgE treatment currently available on the market has therefore been explored in several studies as a possible treatment for AD. Like omalizumab, apheresis has also been shown to exert an immunomodulatory response by stimulating regulatory T cells and normalising the Th1/Th2 immune balance.^82^ However, a non-IgE mediated component of chronic skin inflammation may exist in patients with AD. It is possible that a subset of patients who are sensitized only to the T cell epitope may not show a clear response to anti-IgE therapies.^91^

Results of the efficacy of omalizumab in AD are inconclusive. The limited number of small, randomised controlled studies, few uncontrolled prospective and retrospective reports, as well as multiple case series and reports observed varying degrees of omalizumab efficacy in AD. Strong placebo responses in AD may also play a critical role in the inconclusiveness of efficacy in placebo-controlled studies. In addition, many case series and reports are published on the basis of extraordinary and interesting results, leading to a publication bias that further complicates the ambivalent role of IgE and efficacy of anti-IgE therapy in AD. Nevertheless, results from controlled studies such as the ADAPT study have shown encouraging outcomes that need to be replicated in a larger trial setting.^30^ Observational studies and uncontrolled case series show mostly favourable results for the use of omalizumab in the treatment of AD. The presence of extremely high levels of serum IgE in patients is common, and usually has a correlation with the severity of the disease.^18^^,^^92^ This may be a confounding factor influencing the efficacy of anti-IgE therapy. Large variations in serum levels of IgE, ranging from values considered normal to over 20,000 IU/mL, and the limitation of the approved maximum dose of omalizumab may well be the reason for the spectrum of outcomes. Variable duration of treatment, ranging between 8 and 168 weeks, and dosing frequency of omalizumab used in different studies may also be potential confounding factors. Consistent outcomes indicating a stronger effect in AD patients with lower levels of serum IgE also suggest that a subgroup of patients may benefit from omalizumab therapy. Furthermore, it would be of interest to explore the effect of an anti-IgE treatment in intrinsic AD and involvement of IgE auto-antibodies, similar to that observed in patients with CSU.^93^^,^^94^ Studies that showed the safety of omalizumab in AD deemed the therapy well tolerated, reporting either few or no side effects, with no report of a differential safety profile between different populations e.g. children, adults, women, elderly, patients with co-morbidities, etc. Safety of omalizumab reported in patients is not different from that reported in studies for other therapeutic indications. Persistence of AD in 20% of patients into adulthood also requires therapeutic approaches to be safe during reproductive age and during pregnancy and lactation.^95^ No data exist showing the safety of omalizumab in this special population with AD. However, the EXPECT study showed no evidence of an increased risk of major congenital anomalies with omalizumab in 250 pregnant women treated with anti-IgE therapy.^96^ Ligelizumab, the second generation anti-IgE antibody, which is currently under development, has shown promising results in patients with CSU, but was unable to demonstrate significant improvement in symptoms of asthma versus placebo.^97^ This may indicate that ligelizumab has a slightly different mechanism of action or drug distribution than omalizumab. Given that ligelizumab has demonstrated a 50-fold higher affinity for IgE compared with omalizumab, it would be interesting to see if ligelizumab can demonstrate conclusive efficacy in AD.^98^

Unlike anti-IgE treatment, response to apheresis is not dependent on baseline levels of serum IgE.^82^^,^^83^ Nevertheless, a stronger clinical response was demonstrated in patients with very high levels of IgE.^86^ The efficacy of apheresis in patients is yet another validation of the central role of IgE in this disease. Unfortunately, the benefit of therapy does not last long after the discontinuation of the procedure. Combining apheresis and anti-IgE treatment with omalizumab may serve as a comprehensive therapeutic approach for patients with severe refractory AD with elevated levels of IgE.^29^^,^^72^ The possibility of stabilising levels of IgE through the combination of the 2 therapies is an exciting prospect. Cyclic treatment of the combination may also have to consider possible removal of omalizumab by apheresis if the treatment cycles are not separated optimally. Optimising cycles of apheresis and anti-IgE therapy for individual patients, based on their baseline IgE levels, may help personalise the combination for better outcomes. However, the limitation of availability and cost limits the applicability of these treatment regimen to only a few centres worldwide.

Identifying AD phenotypes that can be responsive to different treatments may be an effective approach to management. Hotze et al. demonstrated a complete lack of response to omalizumab in patients with FLG mutations.^35^ However, Holm et al refuted this observation in their study in just three patients.^42^ Patients with lower levels of serum IgE have shown better responses to omalizumab than those with high or extremely high serum IgE.^32^^,^^35^^,^^37^, ^38^, ^39^^,^^44^^,^^46^^,^^48^^,^^55^^,^^56^^,^^61^^,^^62^^,^^64^ Even in asthma, omalizumab needs to attain a target free-IgE level of ≤10 IU/mL or ≤25 ng/mL to achieve a response. Future dedicated and well conducted studies may implement screening criteria based on distinct AD clusters and endotypes to investigate potential responders to therapies.

There is still a large unmet need for treating patients with AD. Future studies with omalizumab in AD need to answer some unsettled questions such as: Could omalizumab be effective in AD patients with high IgE levels by optimising dosage and dose regimen? Are there different patient populations that respond well to targeting IgE, and would it be of interest to explore a cluster/phenotype-specific response with omalizumab? Could apheresis or other concomitant treatments help facilitate the beneficial effects of omalizumab, and could a combination of different therapies be optimised as a management approach for AD patients with very high serum IgE non-responsive to standard therapies? Would a higher affinity anti-IgE, such as ligelizumab, be effective? Planned studies, with well-defined study populations, on targeting IgE in AD are required to address these unresolved questions.

## Conclusions

IgE seems to play a role in the pathogenesis of AD. Targeting IgE may represent an effective treatment option for many patients with AD. This was evident from numerous studies, which demonstrated the benefits of omalizumab treatment and IgE plasma apheresis. However, some studies investigating the use of omalizumab in AD have been inconclusive, which provokes the notion that IgE may be an epiphenomenon biomarker rather than a pathogenetic factor. This concept may be disputed considering that in many studies, the dose of omalizumab used was low compared with the markedly elevated patient IgE levels, the small and heterogeneous populations studied, and the evidence that IgE seems to play a significant role in majority of AD patients with IgE targeting treatments showing efficacy. Despite its well-known safety profile, established over 15 years of clinical use, no dedicated Phase III study targeting IgE by omalizumab has been conducted in patients with AD. There is a need for dedicated Phase II and III studies with an appropriate AD population and robust study design, which can effectively compensate for a high placebo effect, to systematically investigate omalizumab as a potential therapy for AD and the role of IgE in the disease.

## Abbreviations

AD, atopic dermatitis; CSU, chronic spontaneous urticaria; DFPP, Double-filtration plasmapheresis; DLQI, dermatological life quality index; FLG, filaggrin gene; IGA, investigators' global assessment; IgE, Immunoglobulin E; OX40L, tumour necrosis factor receptor superfamily member 4; QoL, quality of life; SCORAD, scoring atopic dermatitis; TARC, thymus and activation regulated chemokine; Th2, T helper 2 cell; TPE, Therapeutic plasma exchange.

## Declaration of competing interest

**Andreas Wollenberg** has received grants, personal fees or nonfinancial support from 10.13039/100006483Abbvie, Almirall, 10.13039/501100010558Beiersdorf, Bioderma, 10.13039/100010795Chugai, Galapagos, 10.13039/501100009754Galderma, Hans Karrer, Leo Pharma, Eli Lilly, L'Oreal, Maruho, 10.13039/501100004628MedImmune, 10.13039/100004336Novartis, 10.13039/100004319Pfizer, 10.13039/100013226Pierre Fabre, 10.13039/100009857Regeneron, 10.13039/501100004286Santen and Sanofi-Aventis.

**Jean-Philippe Lacour** has received grants/research support as an investigator and honoraria, advisory board, or consulting fees from 10.13039/100006483AbbVie, BMS, 10.13039/100008349Boehringer Ingelheim, 10.13039/100006436Celgene, 10.13039/100013988Dermira, 10.13039/501100009754Galderma, Janssen, 10.13039/100004312Eli Lilly and Company, Leo-Pharma, 10.13039/100004334Merck, 10.13039/100004336Novartis, 10.13039/100009857Regeneron, 10.13039/100004337Roche, and 10.13039/100004339Sanofi.

**Simon Francis Thomsen** has been a paid speaker, served on advisory boards and received research support from 10.13039/100006483Abbvie, Almirall, 10.13039/100006436Celgene, Eli Lilly, GSK, Janssen, Leo Pharma, 10.13039/100004336Novartis, 10.13039/100013226Pierre Fabre, 10.13039/100004337Roche, 10.13039/100004339Sanofi and 10.13039/100011110UCB.

**Xavier Jaumont** and **Slawomir Lazarewicz** are permanent employees of Novartis Pharma AG.
